# Augmented baseplates in reverse shoulder arthroplasty: a systematic review of outcomes and complications

**DOI:** 10.1016/j.xrrt.2022.08.008

**Published:** 2022-10-01

**Authors:** Ramesh B. Ghanta, Ellen L. Tsay, Brian Feeley

**Affiliations:** Department of Orthopedic Surgery, University of California San Francisco, San Francisco, CA, USA

**Keywords:** Augmented baseplates, rTSA, Shoulder arthroplasty, Glenoid wear, RSA, Posterior augments

## Abstract

**Background:**

Glenoid wear secondary to primary osteoarthritis or rotator cuff arthropathy is an obstacle commonly encountered by surgeons performing reverse shoulder arthroplasty, with numerous techniques devised to address this finding. The most recent of such techniques is the introduction of augmented glenoid baseplates to fill these glenoid defects. The objectives of this systematic review are to analyze clinical outcomes of augmented baseplates in patients with glenoid wear, including pain, range of motion, patient-reported functional scores, radiographic outcome measures, complication rates, and revision rates.

**Methods:**

Three online databases (Ovid Medline, EMBASE, Pubmed) were searched for studies publishing clinical and functional outcomes of augmented baseplates in primary reverse shoulder arthroplasty. Findings were aggregated and frequency-weighted means of these variables were calculated when applicable.

**Results:**

Seven studies comprising 810 patients were included in this review. The mean patient age was 72.1 ± 8.1 years with an average follow-up time of 41.4 months. Frequency-weighted means of improvement in forward elevation, abduction, and active external rotation were 53°, 47°, and 19°, respectively. Patients experienced American Shoulder and Elbow Surgeons, Simple Shoulder Test, and Constant score improvements of 45.9, 5.9, and 33.7, respectively. Pooled complicated rate was 6.4%, with 10 cases of baseplate loosening and 3 cases of instability. Five (0.6%) patients required reoperation. Subdividing among augment type (posterior, superior, posterosuperior), there were no apparent differences in outcomes or complication rates between directional augments.

**Conclusion:**

This systematic review demonstrates that augmented baseplates for reverse shoulder arthroplasty provide positive outcomes both clinically and functionally at early follow-up. Complications are within an acceptable range for primary reverse shoulder arthroplasty, with a low rate of revision. Augmented baseplates should serve as a viable option for surgeons seeking to address glenoid wear during reverse shoulder arthroplasty.

Glenoid wear is an obstacle commonly encountered by surgeons performing reverse total shoulder arthroplasty (RTSA).^16^ Primary osteoarthritis of the glenohumeral joint affects 94% of women and 85% of men over the age of 80, and may lead to posterior humeral subluxation and posterior glenoid wear.^9^ Similarly, rotator cuff arthropathy can lead to superior glenoid wear via superior humeral head migration. In a United States population which is rapidly aging and becoming more active leading to a considerable increase in the number of RTSA procedures, addressing this bony erosion is crucial in order to prevent complications, which include baseplate loosening and/or instability, soft tissue imbalance, and scapular notching.^22^

Numerous techniques for addressing glenoid wear have been developed in recent years. The most simple and cost-efficient procedure is eccentric reaming, in which the glenoid is asymmetrically reamed in order to produce a smooth, symmetric glenoid for baseplate implantation.^2^ While this can correct smaller degree defects, asymmetric reaming is not commonly used for more advanced glenoid wear due to concern about removing too much bone. Another option is glenoid bone grafting, which limits the amount of bone lost due to reaming but carries risks of graft failure to incorporate and graft resorption, and remains technically challenging.^13^^,^^18^^,^^21^

A more recent surgical advance is the utilization of metal-backed augmented baseplates to fill glenoid defects.^6^^,^^8^^,^^10^^,^^11^^,^^23^^,^^25^ It is thought that these augmented baseplates offer the ability to correct large glenoid defects without the drawbacks of reaming too much bone, as with eccentric reaming, or risking graft non-incorporation or resorption, as with bone grafting.^15^ Since their introduction to the US market in 2011, augmented baseplates have shown considerable promise at both short- and mid-term follow-up of 2 to 4 years in several isolated studies. However, to our knowledge there are no systematic reviews which aggregate existing evidence to analyze their outcomes. The objectives of this systematic review are to analyze clinical outcomes of augmented baseplates in patients with glenoid wear, including pain, range of motion (ROM), patient-reported functional scores, radiographic outcome measures, complication rates, and revision rates. We hypothesized that augmented baseplates provide improved outcomes and acceptable complication rates compared to standard baseplates or glenoid bone grafting, positioning them as viable options for surgeons performing primary reverse shoulder arthroplasty in shoulders with glenoid wear.

## Materials and methods

**Figure 1:**
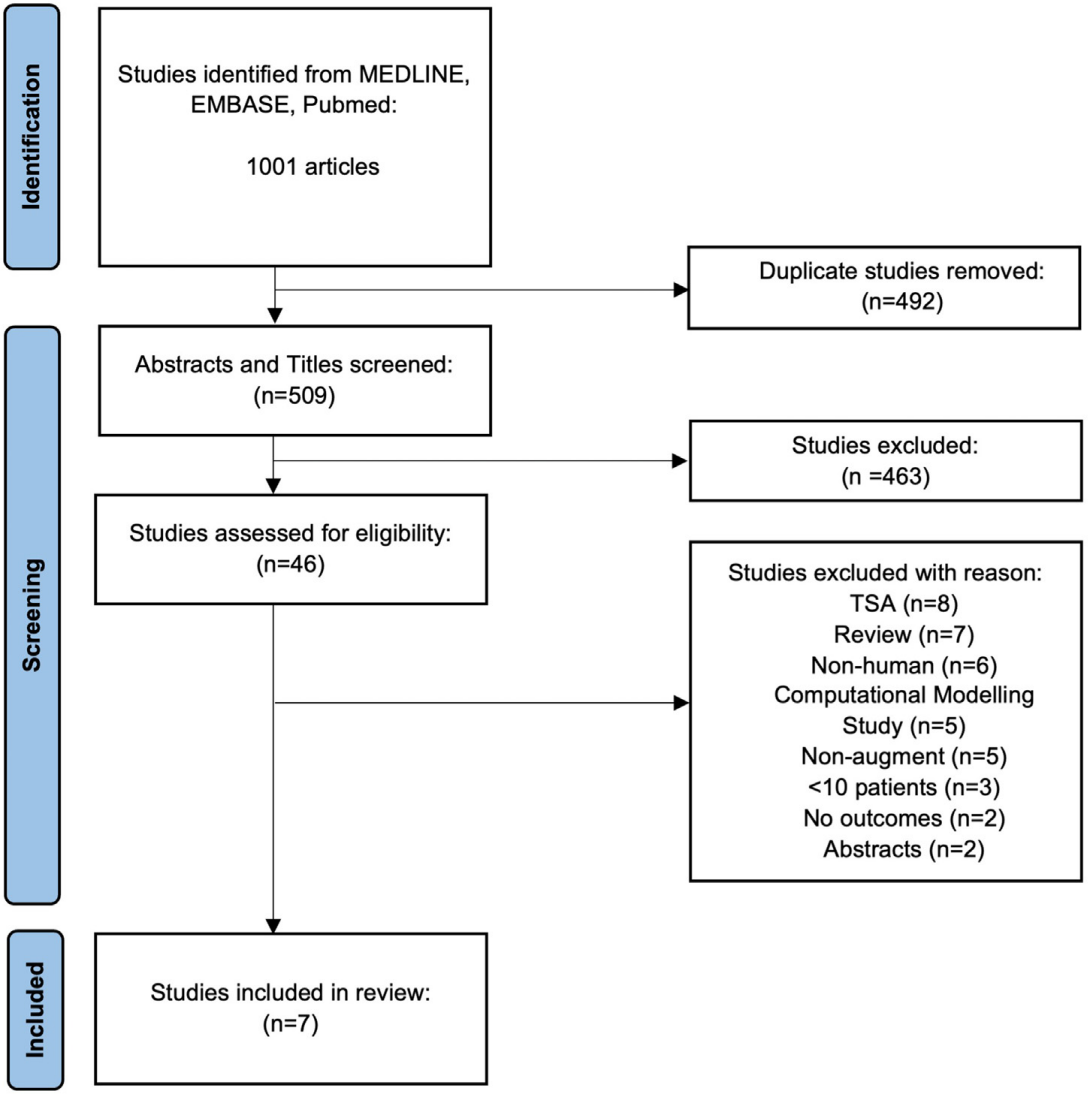
PRISMA flow diagram for study selection and inclusion. *PRISMA*, Preferred Reporting Items for Systematic Reviews and Meta-Analyses.

This systematic review was conducted utilizing the Preferred Reporting Items for Systematic Reviews and Meta-Analyses guidelines (Fig. 1).

### Search strategy

Three online literature databases (Ovid MEDLINE, EMBASE, Pubmed) were searched from database inception to October 4, 2021 for articles that addressed reverse shoulder arthroplasty with utilization of an augmented baseplate. To sufficiently capture all relevant studies, a combination of search terms including reverse shoulder arthroplasty, reverse shoulder, shoulder arthroplasty, RSA, RTSA, augment, augmented glenoid, and augmented baseplate were utilized and supplemented with free text. This search strategy is further elaborated upon in Supplementary Figure S1.

### Inclusion and exclusion criteria

Both inclusion and exclusion criteria were determined a priori. Inclusion criteria were defined as patients aged 18 years or older undergoing primary reverse shoulder arthroplasty, at least 10 patients in the study receiving augmented baseplate, minimum of 12 months of follow-up, and English-language studies. Exclusion criteria include nonhuman studies, meta-analyses, systematic reviews, opinion pieces, and studies in which no distinction was made between the outcomes of augmented baseplates versus other techniques.

### Study screening and selection

All articles were screened and data extraction was performed independently by 2 authors (R.G, E.T). In the abstract and title screening stage, authors were blinded to all information except the abstract/title (data extraction software used by the authors relied upon the abstract and title as primary identifiers for each study) and any article in which there was no consensus between the two reviewers was included to ensure all possible studies were captured. In the full-text review stage, any article in which there was disagreement was adequately discussed and a consensus was reached between the two reviewers. In addition, during full-text screening, reference lists of all included studies were scanned to assess for any additional studies which could meet the inclusion criteria. The Methodological Index for Non-Randomized Studies (MINORS) criteria were applied to all included studies to assess the quality of each study.

### Outcomes collection

Outcomes were collected independently by two authors (R.G, E.T) and finalized after discussion to ensure that every study outcome was properly reported. The primary outcome of interest was the improvement in ROM. Secondary outcomes of interest were the improvement in various patient-reported outcomes. Internal rotation was scored and assessed in the same manner in the majority of studies by the following scoring system: 0[degrees] = 0, hip = 1, buttocks = 2, sacrum = 3, L5-L4 = 4, L3-L1 = 5, T12-T8 = 6, and T7 or higher = 7. In addition, complications and degree of deformity correction were also collected and analyzed, when available.

### Statistical analysis

All outcome measures were pooled, and in applicable situations, frequency-weighted means were calculated. These means adequately represent the mean for each study weighted by the number of patients in each study.

## Results

### Search

The initial search yielded 509 articles when duplicates were removed. After abstract screening by two independent reviewers (R.G, E.T), 46 articles progressed to full-text review. Following full-text review of these studies, 7 manuscripts were included for final analysis.

### Study characteristics

Of the 7 manuscripts included in this review, all were retrospective in nature. Four were Level IV case series while 3 were Level III comparative studies, in which the comparison group was either patients receiving bone graft, a standard baseplate, or separate augment types (posterior vs. superior). A summary of the studies is depicted in Table I.

**Table I:** Summary of included studies.

Study	Study design	No. of patients	Mean age, y	Mean FU mo	Augment characteristics	Clinical outcomes					Functional outcomes						Complications	MINORS score
						Forward flexion	Abduction	Passive ER	Active ER	IR	ASES	SST	SPADI	UCLA	Constant	VAS		
Jones et. al^6^	Level III Retrospective Comparative Study	39	72.1±8.5	28.3±5.7	24 PAB^a^ 15 SAB^b^	Y	Y	Y	Y	Y	Y	Y	Y	Y	Y	N	0	15
Wright et. al^25^	Level II Retrospective Comparative Study	39	72.1±8.5	28.3±5.7	24 PAB 15 SAB	Y	N	Y	Y	Y	Y	Y	Y	Y	N	N	0	16
Michael et. al^15^	Level IV Retrospective Case Series	139	-	-	50 PAB 22 SAB 67 PSAB^c^	Y	N	N	Y	N	Y	N	N	N	Y	N	16 (11.5%)	4
Liuzza et. al^11^	Level IV Retrospective Case Series	68	73.5±8.7	39.6±13.7	33 SAB 35 PSAB	Y	Y	N	Y	Y	Y	Y	Y	Y	Y	N	5 (7.4%)	10
Virk et. al^23^	Level IV Retrospective Case Series	67	71.6±7.8	39.7±15.4	PAB	Y	Y	N	Y	Y	Y	Y	Y	Y	Y	N	3 (4.5%)	11
Gulotta et. al^4^	Level III Retrospective Comparative Study	414	72.0	47.1±23.1	190 PAB 83 SAB 141 PSAB	Y	Y	N	Y	Y	Y	Y	Y	Y	Y	N	7 (3.7%) PAB 3 (3.6%) SAB 4 (2.8%) PSAB	17
Kirsch et. al^10^	Level IV Retrospective Case Series	44	72±6	16.2 (12-27)	15 Half-wedge 29 Full-wedge	Y	N	Y	N	Y	Y	Y	N	N	N	Y	14 (31.8%)	12

*FU*, follow-up; *ER*, external rotation; *IR*, internal rotation; *ASES*, American Shoulder and Elbow Surgeons; *SST*, Simple Shoulder Test; *SPADI*, Shoulder Pain and Disability Index; *VAS*, visual analog scale; *MINORS*, Methodological Index for Non-Randomized Studies.

a Posteriorly augmented baseplate.

b Superiorly augmented baseplate.

c Posterosuperior augmented baseplate.

The mean number of patients in each study was 115.7 (range 39-414), with a total of 810 pooled patients. In 671 (82.8%) patients with gender data available, 51.4% were male and 48.6% were female. In studies with available data, the mean age of patients was 72.1±8.1 years. The average follow-up time was 41.4 months. In 93.4% of patients, the Exactech Equinoxe was utilized, with 331 (46.3%) of these patients receiving a posteriorly augmented baseplate (PAB), 153 (21.9%) receiving a superiorly augmented baseplate (SAB), and 243 (31.2%) patients receiving a posterior-superiorly augmented baseplate (PSAB). The remaining 6.6% of patients received a Tornier Perform+ implant, with 34% of these patients receiving a half-wedge implant and 66% receiving a full-wedge implant.

In the 4 non-comparative studies, the mean MINORS score was 9.3 (range 4-12) out of maximum 16 points. In the 3 comparative studies, the mean MINORS score was 16 (range 15-17) out of maximum 24 points.

### Clinical outcomes

**Figure 2:**
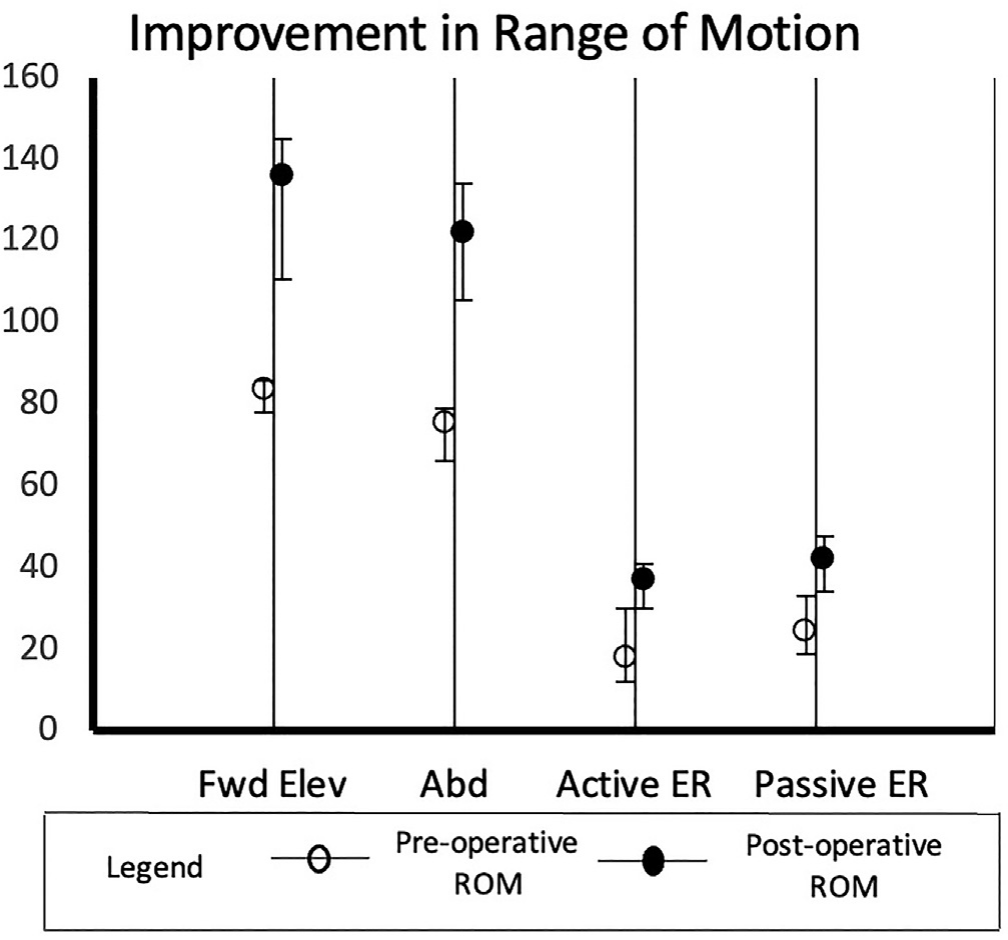
Mean improvement in range of motion postoperatively (*Fwd Elev*, forward elevation; *Abd*, abduction; *active ER*, active external rotation; *passive ER*, passive external rotation).

All studies measured clinical outcomes via ROM assessment both preoperatively and at most recent clinical follow-up. The improvement in ROM is graphically represented in Figure 2. Pooling the results of all studies, frequency-weighted mean of forward elevation improved from 84° (range 78-86) to 136° (range 111-145), abduction improved from 76° (range 66-79) to 122° (range 106-134), active external rotation improved from 18° (range 12-30) to 37° (range 30-41). Internal rotation was assessed on a 7-point scale in the same manner for 627 patients and improved from 3.0 (range 2.4-3.7) points to 4.3 (range 4.12-4.8) points. Three studies comprising 122 patients also reported changes in passive external rotation, which improved from 24° (range 19-33) to 42° (range 34-48).

Subdividing among each augment type, PAB patients improved in forward elevation from preoperative frequency-weighted mean of 85 (range 85-85.4) to 142 (range 133-145), abduction from 75 (range 74-79) to 126 (range 124-134), passive external rotation from 24 (n = 1 study) to 46, active external rotation from 16 (range 12-16) to 36 (range 30-38), and internal rotation from 2.8 (range 2.4-2.9) to 4 (4.1-4.3) points. SAB patients improved in forward elevation from 83 (range 77-86) to 128 (range 111-132), abduction from 73 (range 66-75) to 113 (range 108-115), passive external rotation from 33 (n = 1 study) to 48, active external rotation from 22 (range 16-30) to 38 (range 33-40), and internal rotation from 3.5 (range 3-3.7) to 4.6 (range 4.4-4.8) points. PSAB patients improved in forward elevation from 82 (range 78-83) to 133 (range 132-133), abduction from 78 (range 75-78) to 126 (124-131), active external rotation from 19 (range 17-20) to 39 (range 35-41), and internal rotation from 3.1 (range 2.6-3.2) to 4.4 (4.2-4.4) points. Outcomes subdivided by augment type are represented in Table II.

**Table II:** Outcomes and complications subdivided by augment type.

Variable	Posterior augment		Superior augment		Posterosuperior augment	
	Preoperative	Postoperative	Preoperative	Postoperative	Preoperative	Postoperative
Forward elevation	85.3 (85-85.4)	142.4 (133-145)	83 (77-85.8)	128 (110.7-132.2)	81.8 (78-82.6)	132.7 (132-132.7)
Abduction	75.4 (74.1-79)	126.3 (123.6-134)	72.5 (66-75.1)	112.8 (108-114.7)	77.7 (75-78.4)	125.7 (124.4-131)
Passive external rotation	23.7	45.9	33.2	47.7	-	-
Active external rotation	15.9 (12-16.3)	36 (30-38.2)	22 (16-30)	37.6 (33.1-39.7)	19.1 (17-20.4)	39 (35-41)
Internal rotation	2.8 (2.4-2.9)	4 (4.1-4.3)	3.5 (3.3-3.7)	4.6 (4.4-4.8)	3.1 (2.6-3.2)	4.4 (4.2-4.4)
ASES	37.7 (36.7-39.9)	87.3 (86.8-89)	40.4 (35-41.5)	80.1 (73.4-82.7)	36.4 (33.8-37.2)	81.8 (80-83)
SST	4.0 (3.5-4.1)	10.6 (9.8-11)	4.5 (4.2-4.9)	9.7 (8.7-9.8)	4.2 (3.6-4.3)	9.9 (9.8-9.9)
Constant	36.1 (34-36.9)	72.5 (70-75)	36.7 (32-38.5)	64.9 (59-66.9)	35.3 (33.6-36.4)	68.8 (66-70.2)
SPADI	81.9 (73.2-82.9)	17.4 (16-20.2)	78.9 (74.8-82.6)	25.8 (23.6-39.4)	85.1 (84.2-88.9)	24.6 (24.1-24.7)
UCLA	13.7 (13.2-14)	31.4 (30.6-32)	13.7 (13.3-14.2)	29.8 (27.9-30.3)	13.6	30.2
Complications	14 (4.0%)		9 (5.9%)		17 (7.0%)	

Values represented as frequency-weighted mean with range in parentheses.

*ASES*, American Shoulder and Elbow Surgeons score; *SST*, Simple Shoulder Test; *SPADI*, Shoulder Pain and Disability Index.

### Functional outcomes

**Figure 3:**
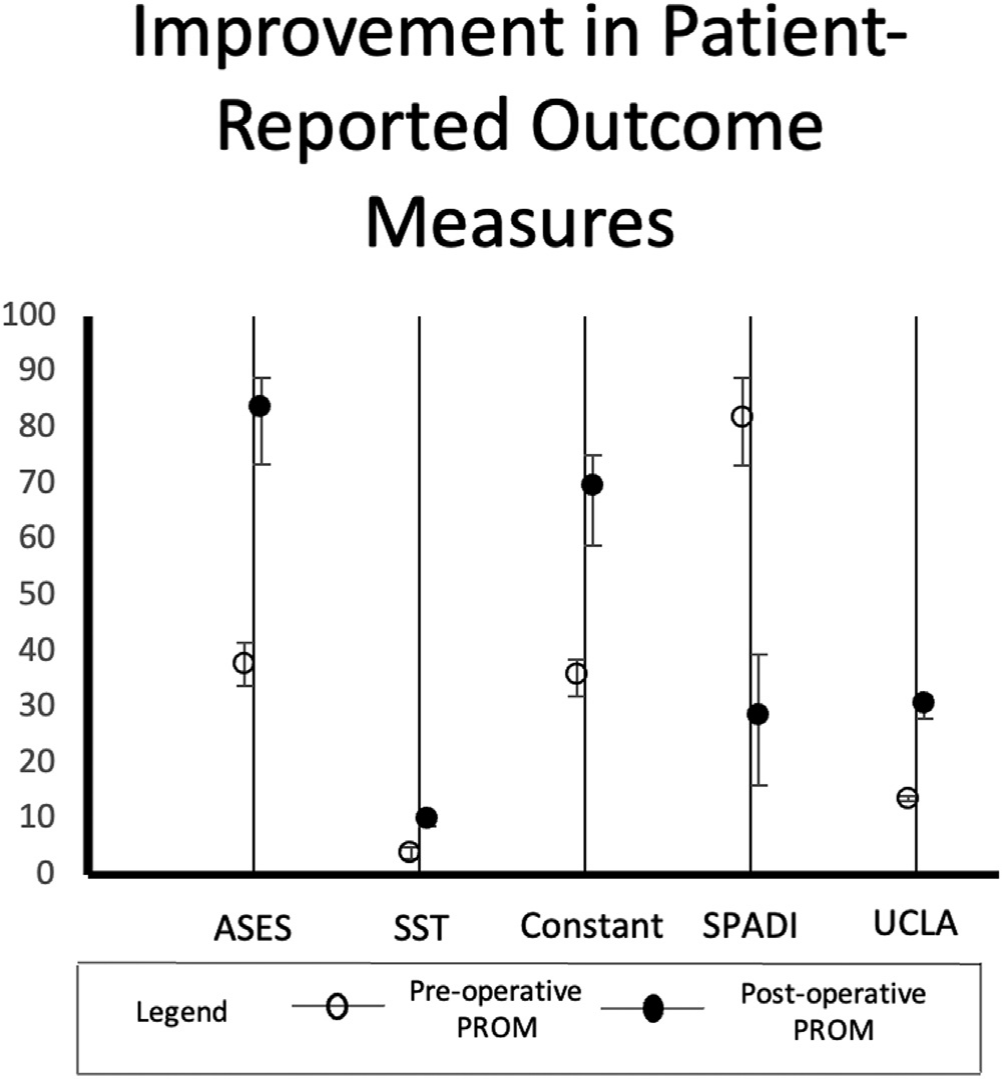
Mean improvement in patient-reported outcome measures postoperatively.

All seven studies assessed functional outcomes via a variety of patient-reported outcome measures (PROMs) collected both preoperatively and at most recent clinical follow-up. The improvement in each functional outcome metric is graphically represented in Figure 3.

The American Shoulder and Elbow Surgeons (ASES) score was assessed in all seven studies. The frequency-weighted mean of these 810 patients improved from 37.8 (range 33.8-41.5) to 83.7 (range 73.4-89). In addition, 4 studies comprising 593 patients calculated the percentage of patients who exceeded the minimal clinically important difference (MCID) and those who exceeded substantial clinical benefit (SCB). In these studies, the frequency-weighted mean of patients who exceeded MCID was 94.6% and those who exceeded SCB was 84.2%. Subdividing among augment types, PAB patients improved from 37.7 (range 36.7-39.9) to 87.3 (range 86.8-89), SAB patients improved from 40.4 (range 35-41.5) to 80.1 (range 73.4-82.7), and PSAB patients improved from 36.4 (range 33.8-37.2) to 81.8 (range 80-83).

The Simple Shoulder Test (SST) score was assessed in six studies. The frequency-weighted mean of these 671 patients improved from 4.1 (range 2.9-4.9) to 10.0 (range 8.7-11). In the four studies comprising 593 patients who calculated the percentage of patients exceeding MCID and SCB, the frequency-weighted mean of patients who exceeded MCID was 88.9% and those who exceeded SCB was 77.2%. Subdividing among augment types, PAB patients improved from 4.0 (range 3.5-4.1) to 10.6 (range 9.8-11), SAB patients improved from 4.5 (range 4.2-4.9) to 9.7 (range 8.7-9.8), and PSAB patients improved from 4.2 (range 3.6-4.3) to 9.9 (range 9.8-9.9).

The Constant score was assessed in six studies. The frequency-weighted mean of these 766 patients improved from 35.9 (range 32-38.5) to 69.6 (range 59-75). In the three studies comprising 549 patients who calculated the percentage of patients exceeding MCID and SCB, the frequency-weighted mean of patients who exceeded MCID was 98.2% and those who exceeded SCB was 86.3%. Subdividing among augment types, PAB patients improved from 36.1 (range 34-36.9) to 72.5 (range 70-75), SAB patients improved from 36.7 (range 32-38.5) to 64.9 (range 59-66.9), and PSAB patients improved from 35.3 (range 33.6-36.4) to 68.8 (range 66-70.2).

The Shoulder Pain and Disability Index score was assessed in five studies. The frequency-weighted mean of these 627 patients improved from 81.8 (range 73.2-88.9) to 28.4 (range 16-39.4). In the three studies comprising 549 patients who calculated the percentage of patients exceeding MCID and SCB, the frequency-weighted mean of patients who exceeded MCID was 92.2% and those who exceeded SCB was 77.1%. Subdividing among augment types, PAB patients improved from 81.9 (range 73.2-82.9) to 17.4 (range 16-20.2), SAB patients improved from 78.9 (range 74.8-82.6) to 25.8 (range 23.6-39.4), and PSAB patients improved from 85.1 (range 84.2-88.9) to 24.6 (range 24.1-24.7).

The University of California Los Angeles (UCLA) score was assessed in five studies. The frequency-weighted mean of these 627 patients improved from 13.7 (range 13.2-14.2) to 30.6 (range 27.9-32). In the three studies comprising 549 patients who calculated the percentage of patients exceeding MCID and SCB, the frequency-weighted mean of patients who exceeded MCID was 94.5% and those who exceeded SCB was 88.4%. Subdividing among augment types, PAB patients improved from 13.7 (range 13.2-14) to 31.4 (range 30.6-32), SAB patients improved from 13.7 (range 13.3-14.2) to 29.8 (range 27.9-30.3), and PSAB patients improved from 13.6 (n = 1 study) to 30.2.

The visual analog scale was assessed in one study. The mean of these 44 patients improved from 6.3 preoperatively to 0.9 postoperatively.

### Complications

Among all studies with 810 pooled patients, there were 52 (6.4%) reported complications. With respect to fractures, there were 8 acromial stress fractures, 5 peri-prosthetic fractures, 3 postsurgical traumatic fractures, 1 glenoid fracture, and 1 scapular neck fracture. In the 3 studies with acromial stress fractures as complications, 6 patients (75%) had Favard E2 or E3 glenoids and 5 (63%) fractures occurred using the Tornier Perform+ system. There were 10 cases of baseplate loosening, 3 cases of instability, and 1 humeral liner dissociation. In addition, there were 2 patients who developed postoperative hematomas, 2 patients with superficial infection requiring antibiotics, and 1 case of proximal median neurapraxia. Five (0.6%) patients required reoperation.

### Deformity corrections

One study examined radiographic outcomes via measurement of the acromiohumeral distance and the lateral humeral offset. In this study, patients received a Tornier Perform+ implant with either half-wedge or full-wedge augment. Acromiohumeral distance improved from 8.9 mm preoperatively to 34 mm postoperatively. In this study, lateral humeral offset also improved from 34 mm preoperatively to 12.7 mm postoperatively.

## Discussion

In this systematic review consisting of 810 pooled patients, we found that augmented baseplates for primary reverse shoulder arthroplasty provide excellent clinical and functional outcomes at follow-up ranging from 16 to 47 months with an acceptable complication rate. Postoperative ROM improved in all planes of motion and all patient-reported outcomes demonstrated considerable improvement with a pooled mean complication rate of 6.4%. These findings suggest that augmented baseplates provide excellent clinical and functional outcomes and can serve as alternatives to standard baseplates or glenoid bone grafting, although further studies are required to directly compare these treatment modalities.

The primary outcome of interest, collected in all seven studies, was improvement in ROM from preoperative baseline. In our pooled patient population, forward flexion increased by 53°, abduction by 47°, active external rotation by 19°, and internal rotation by 1.2 points. The existing literature analyzes the effect on ROM after using an alternative technique to address glenoid erosion in RTSA: bone grafting. In a systematic review of this literature by Paul et al that compiles outcomes from 276 patients, there was a mean improvement in forward flexion of 64°, abduction of 63°, and external rotation of 13° after RTSA with bone grafting.^18^ While our study found a lower absolute degree of improvement in forward flexion and abduction in patients with augmented baseplates compared to these patients with bone grafting, it is possible that this is in part due to differing surgical indications for each technique that confound the absolute degree of improvement seen, with augmented baseplates used for more severe and difficult-to-address glenoid defects. Given that augmented baseplates have an overall positive impact on ROM for patients with severe glenoid wear, they could play a useful role in revision settings where humeral head autograft may not be readily available.

In addition to ROM outcomes, all studies looked at a variety of different PROMs in an effort to capture the subjective, perceived benefit of augmented baseplates for RTSA. Every study included the ASES score, many included the SST and Constant scores, and a smaller subset of the studies included the Shoulder Pain and Disability Index and UCLA scores. Our patients experienced an improvement of 45.9 in their ASES score, compared to improvements of 33.8-44 for published studies utilizing bone grafting.^7^^,^^12^^,^^13^^,^^21^^,^^24^ Our patients also experienced 5.9 and 33.7 point improvements in SST and Constant scores, respectively, compared to ranges of 2.4-5.9 and 20.6-61 point improvements reported in the literature for bone grafting.^1^^,^^3^^,^^5^^,^^7^^,^^12^^,^^13^^,^^21^^,^^24^ Several of our studies also measured the percentage of patients who met the threshold for MCID and SCB, which were consistently above 90% and 80%, respectively. These findings illustrate that the vast majority of patients experienced subjective improvements in pain, level of function, and clinical outcome after implantation with an augmented baseplate, and in many cases these improvements were statistically significant.

With respect to complications, there was a pooled 6.4% rate of complications among our patient sample. Subdividing these complications into categories, 34.6% of the total complications were a result of an intraoperative or postoperative fracture, 19.2% were a result of baseplate loosening, 5.8% were due to instability, and 3.8% were from postoperative hematomas or superficial infection each. Further examining postoperative fractures, 1 study which closely analyzed 5 patients who developed acromial stress fractures could not identify any preoperative risk factors for the development of this complication other than decreased glenoid retroversion.^10^ Acromial stress reactions and fractures are likely under-reported within the literature and thus, more data are needed to determine how augmented baseplates related to this pathology. The remaining complications were due to a number of isolated events, including humeral liner dissociation and median neurapraxia. There was a 0.6% reoperation rate. In this review of RTSA with augmented baseplates, we found a lower complication rate than those reported in the existing literature for both primary RTSA, as well as RTSA with glenoid bone grafting.^4^ In a recent large international database study of primary RTSA by Parada et al, the authors found a complication rate of 8.9% with a revision rate of 2.5% in primary RTSA.^17^ In a study of glenoid bone grafting in primary RTSA, Malahias et al found an all-cause reoperation rate of 3.5%.^14^ They also looked at rates of specific complications and found an aseptic loosening rate of 3.1%, a periprosthetic fracture rate of 4.8%, and a 0.9% infection rate. These results suggest that augments have lower rates for specific complications. For example, a biomechanical study by Roche et al found that, when comparing eccentric reaming with a standard baseplate to superior augments, there was no difference in fixation between the two methods but augments conserved significantly more glenoid bone, which could potentially decrease baseplate loosening.^20^ Meanwhile, bone grafting, while able to correct more severe defects, also carries the risk of graft non-incorporation and donor site morbidity if humeral head autograft is not utilized, complications which can be avoided by the utilization of an augmented baseplate. Within the context of this published literature, it appears that RTSA with augmented baseplates provides low and acceptable complication and reoperation rates while potentially being able to preserve more glenoid bone and eliminate the risks of graft nonunion.

Additionally, we subdivided by specific augment type (posterior, superior, posterosuperior) to analyze outcomes and complications rates for each. In general, posterior augments are indicated in classic cases of shoulder osteoarthritis in which the posterior glenoid is preferentially more worn (ie, Walsh B2 glenoid). Superior augments are utilized in cases of rotator cuff deficiency and rotator cuff arthropathy, in which superior migration of the humeral head leads to preferential wearing the superior glenoid. Posterosuperior augments are typically used in scenarios where a combination of these defects is present or when the surgeon is aiming to tension the posterior rotator cuff.^19^ For each specific augment, ROM improved in all planes of motion and all PROMs indicated improved from preoperative baseline (Table II). Additionally, complication rates were similar, with a rate ranging from 4.0% for posterior augments to 7.0% for posterosuperior augments. While both rates are within the realm of acceptable complication rates for reverse shoulder arthroplasty, it is possible that the complication rate was higher for posterosuperior augments due to the potentially more severe glenoid defects in patients requiring this specific type of augment.

This study does carry some limitations. First, the majority of our included studies were Level III or IV papers, with all studies being retrospective in nature. The overall quality of our included studies was deemed as moderate per the MINORS scale, but one study was deemed as low quality. In addition, two of the studies appeared to analyze an identical patient sample to report their findings, but as we were unable to definitively prove this, both results were included in the pooled patient sample. Multiple studies also utilized the same industry database, which likely also led to duplication of reported data. These findings reflect the need for prospective studies which can directly compare the outcomes and complications of augmented baseplates with other techniques to address glenoid erosion. Also, only 1 study analyzed preoperative glenoid deformity and was thus able to comment on deformity correction by the augmented baseplate. This represents a limitation of the current literature and a gap which should be addressed in further studies.

Additionally, the majority of studies were multicenter, multi-surgeon studies. Differences in surgical indications, patient selection, surgical technique, and postoperative rehabilitation protocols between centers and surgeons could introduce bias. On the other hand, the multicenter nature of this study also increases the generalizability of its findings, as patients had positive outcomes and acceptable complication rates in all studies.

Finally, this study is limited by duration of follow-up, with the longest follow-up reported as 47 months. While these data demonstrate the positive benefits of augmented implants at both early follow-up, it does not prove the long-term survivability of these implants. Since reverse shoulder arthroplasty has a relatively long implant survivorship of up to 10 years, and now that augmented baseplates have been introduced to the US market for 11 years now, there is a need for further studies that evaluate the implants’ long-term outcomes and complications.

## Conclusion

This systematic review demonstrates the promising outcomes when utilizing augmented baseplates to address glenoid wear in reverse shoulder arthroplasty. Patients receiving either posterior, superior, or posterosuperior augments experienced considerable improvement in ROM in all planes at most recent follow-up. Functional outcomes also demonstrated considerable improvement among all patients receiving augmented baseplates. Complications were within an acceptable range, with low rates of baseplate loosening, instability, or reoperation. These findings illustrate that augmented baseplates serve as viable options for surgeons encountering severe glenoid wear during reverse shoulder arthroplasty.

## Disclaimers:

Funding: No funding was disclosed by the authors.

Conflicts of interest: The authors, their immediate families, and any research foundation with which they are affiliated have not received any financial payments or other benefits from any commercial entity related to the subject of this article.
