# Supplementary material for: Augmented baseplates in reverse shoulder arthroplasty: a systematic review of outcomes and complications
Source: JSES Rev Rep Tech. 2022 Oct 1;3(1):37–43. doi: 10.1016/j.xrrt.2022.08.008 (PMC10426546; doi:10.1016/j.xrrt.2022.08.008)
Supplement: Supplementary Figure S1 [file mmc1.pdf]

Summary of Database Search Strategy

Ovid MEDLINE

#	Query	Results
1	(RSA or RTSA or reverse shoulder arthroplasty or reverse shoulder or reverse total shoulder arthroplasty or reverse arthroplasty).mp.	7,920
2	(augment* or augment* baseplate or augment* glenoid).mp.	198,170
3	1 and 2	184

mp=title, abstract, original title, name of substance word, subject heading word, floating sub-heading word, keyword heading word, organism supplementary concept word, protocol supplementary concept word, rare disease supplementary concept word, unique identifier, synonyms

EMBASE

#	Query	Results
1	((((reverse AND shoulder AND arthroplasty OR reverse) AND total AND shoulder AND arthroplasty OR reverse) AND shoulder OR reverse) AND arthroplasty OR shoulder) AND arthroplasty OR rsa OR rtsa	30004
2	augment*	262693
3	(((((reverse AND shoulder AND arthroplasty OR reverse) AND total AND shoulder AND arthroplasty OR reverse) AND shoulder OR reverse) AND arthroplasty OR shoulder) AND arthroplasty OR rsa OR rtsa) AND augment*	589

PUBMED

#	Query	Results
1	(((((((RSA) OR (RTSA)) OR (reverse shoulder arthroplasty)) OR (reverse shoulder)) OR (reverse total shoulder)) OR (reverse arthroplasty))) AND ((augment*))	223
